# Tumor Lysis Syndrome in an Elderly Male With Newly Diagnosed Large Cell B-Lymphoma Despite Prophylactic Treatment

**DOI:** 10.7759/cureus.77958

**Published:** 2025-01-25

**Authors:** Erin S Reid, Hannah Nix, Michael Ibrahim, Andreas Maddux

**Affiliations:** 1 Internal Medicine, Alabama College of Osteopathic Medicine, Dothan, USA; 2 Nephrology, UAB St. Vincent's East, Birmingham, USA

**Keywords:** basic oncology, chemotherapy effect, internal medicine and clinical nephrology, metastatic diffuse large b-cell lymphoma, tumor-lysis syndrome

## Abstract

Tumor lysis syndrome (TLS) is a life-threatening complication that can arise after initiation of cytotoxic chemotherapy in highly proliferative hematological malignancies, such as leukemias or lymphomas. Prophylactic measures, such as allopurinol, rasburicase, and hydration, are commonly initiated in these types of malignancies prior to chemotherapy to prevent TLS. However, in rare instances, TLS can still occur despite the initiation of prophylactic treatment. When TLS develops, the patient can experience various complications, such as kidney injury, muscle fatigue, paralysis, cardiac arrhythmias, and even death. Our case presents an 83-year-old male with newly diagnosed stage IV diffuse large B-cell lymphoma (DLBCL) who developed TLS following chemotherapy despite receiving the standard prophylactic treatment of IV fluids, rasburicase, and allopurinol. This case highlights the importance of identifying signs of TLS and initiating appropriate treatment even in patients receiving prophylaxis.

## Introduction

Tumor lysis syndrome (TLS) can occur in patients undergoing treatment for malignancy, during surgical manipulation, and spontaneously in highly proliferative tumors and can result in life-threatening emergencies. TLS typically occurs after the initiation of cytotoxic chemotherapy due to rapid degeneration and lysis of tumor cells [1]. During the lysis of cells, numerous electrolytes are released, causing major disturbances within the body. The electrolyte and metabolic disturbances that are characteristically associated with TLS include hyperuricemia, hyperkalemia, hyperphosphatemia, and hypocalcemia [2]. Due to these shifts in electrolytes, patients can experience life-threatening problems, including cardiac arrhythmias, renal failure, or death [2]. While TLS can occur with any type of cancer, TLS most commonly occurs with cancers that are highly sensitive to chemotherapy, possess a high tumor burden, or have a high proliferative rate. Due to the fatal complications associated with TLS, prophylaxis treatment is routinely initiated, which includes rasburicase, allopurinol, and IV fluids [3].

This case demonstrates that TLS can rapidly occur even with appropriate prophylactic treatment. Despite administration of rasburicase, allopurinol, and IV fluids, the patient still experienced major metabolic disturbances associated with TLS. Additionally, this case highlights the current limitations of prophylactic treatment for TLS in individuals who have highly advanced and aggressive tumors. These tumors carry a large tumor burden that may impact the ability of the prophylactic management to fully neutralize the body’s response to cytotoxic therapy.

## Case presentation

An 83-year-old male, with a past medical history significant for type 2 diabetes mellitus, hypertension, and hyperlipidemia, presented to the emergency department (ED) due to complaints of generalized weakness, diminished appetite, shortness of breath, and a non-productive cough that had been ongoing for four weeks. He was previously seen at another ED five days prior presenting with the same complaints. A full work-up was completed on the patient in the ED.

Labs revealed an elevated WBC, mild anemia, and an unremarkable BNP (Table 1). Hematology/oncology was consulted due to concern for possible malignancy. The patient was admitted for further studies and imaging. Our team, nephrology, was consulted four days after the patient was admitted due to abnormalities in his electrolytes. Prior to our consultation, the patient had been diagnosed with stage IV diffuse large B-cell lymphoma (DLBCL) and was subsequently initiated on R-CHOP chemotherapy.

**Table 1:** Patient's significant labs during emergency room admission

	Patient Value	Normal Range
WBC	27,900	4,500-11,000 cells/mcL
RBC	3.96	4.20-5.40 million cells/mcL
Hemoglobin	10.4	11.6-14.8 g/dL
Hematocrit	32.3%	34.0%-44.0%
BNP	133	<100 pg/mL

This advanced stage of cancer carries a high tumor burden and an increased risk of TLS upon initiation of chemotherapy. Therefore, the patient was started on aggressive prophylaxis with IV fluids (NaCl 0.9% 250 mL rate, 25 mL/hour), rasburicase (single 6 mg dose), and daily allopurinol (300 mg) prior to initiation of R-CHOP chemotherapy. Despite prophylaxis, the patient developed laboratory findings consistent with TLS (Table 2). Emergent treatment focused on regulating these electrolyte and metabolic abnormalities with insulin glargine, sodium zirconium cyclosilicate (Lokelma), IV NaCl fluids, continued allopurinol doses, and a platelet transfusion. Within 48 hours of treatment, the patient’s electrolyte abnormalities stabilized. The patient was continuously monitored throughout his treatment to ensure his vitals remained stable.

**Table 2:** Electrolyte and metabolic abnormalities within 48 hours of R-CHOP initiation

	Patient Value	Normal Range
Uric Acid	8.3	3.5-7.2 mg/dL
Potassium	5.9	3.5-5.1 mmol/L
Calcium	8.1	8.5-10.2 mg/dL
Phosphorous	4.7	2.8-4.5 mg/dL
Platelets	9,000	140-450 per mcL

## Discussion

TLS is considered an oncologic emergency and is a predictor for overall worse morbidity and mortality in cancer patients [1,2]. It is characterized by a rapid breakdown of cancerous cells usually in response to cytotoxic therapy, such as chemotherapy, but can also occur spontaneously when there is a large tumor burden, high proliferative rate, or high sensitivity to chemotherapy [3]. If a patient develops TLS, they can present with hyperkalemia, hyperuricemia, hyperphosphatemia, and hypocalcemia. Hyperuricemia is one of the hallmarks of TLS and if left untreated can lead to acute kidney injury. Adenosine and guanine are purine-containing nucleic acids within DNA that are released into the serum during cell lysis. Once in the serum, they are broken down into uric acid by xanthine oxidase. Uric acid is water-insoluble in the distal tubules and collecting ducts because of the acidic environment. This results in the buildup of uric acid crystals in the renal tubule, which can cause obstructive nephropathy, compromising glomerular filtration and urine output [2].

Another hallmark of TLS, hyperphosphatemia, can overload the phosphate excretion system within the kidney. This allows excess phosphate to bind calcium within the body, causing secondary hypocalcemia and calcium phosphate deposition throughout the body. Potentially fatal complications of hypocalcemia include seizures or fatal arrhythmias. Additionally, the calcium phosphate crystals can precipitate within the renal tubules resulting in nephrolithiasis [2]. Additionally, TLS can result in hyperkalemia as potassium is released from tumor cells. This can lead to complications such as cardiac arrhythmias and sudden cardiac death [2].

Each of these electrolyte disturbances that result from TLS can be potentially fatal if not identified and promptly treated. Standard protocol for prophylaxis treatment to prevent TLS includes IV fluids, allopurinol, and rasburicase. IV fluids are one of the mainstays of prophylactic treatment and should be given at 2-3 L/m^2^ per day [3]. The length of IV therapy is dependent upon different factors, including tumor burden, type of chemotherapy used, drug sensitivity of the tumor, patient’s ability to drink, and renal function [3]. Just as the length of fluids is dependent upon several varying factors, the type of fluid used is also varied in different patient situations. Isotonic saline is usually the initial fluid of choice, especially in patients who are hyponatremic or volume-depleted [3]. Allopurinol is another medication implemented as a standard prophylactic treatment for TLS. This drug is an analog of hypoxanthine and competitively inhibits xanthine oxidase [4]. This blocks the conversion of hypoxanthine and xanthine into uric acid. Rasburicase, the third component in the prevention of TLS, also works to reduce uric acid levels but relies on a different mechanism than allopurinol. While allopurinol works to decrease uric acid levels over time, rasburicase works to acutely reduce uric acid levels [3]. Rasburicase is a recombinant form of the enzyme urate oxidase [3]. This enzyme catalyzes the oxidation of uric acid to a more water-soluble compound, allantoin, which allows it to be excreted without causing kidney damage [3].

Our case report highlights a patient with DLBCL. This type of cancer is one of the most common lymphoid malignancies in adults in Western areas of the world [5]. It is a rapidly growing and extremely aggressive lymphoma known to have a large tumor load [6]. These characteristics increase the risk of a patient developing TLS despite appropriate prophylaxis as in this case. This case highlights the current limitations of prophylactic treatment for TLS in individuals who have highly advanced and aggressive tumors.

## Conclusions

TLS is an oncological emergency that must be closely monitored and urgently treated due to severe metabolic disturbances within the body. This case report highlights the limitations of current prophylactic regimens for TLS in high-risk cancers. Close monitoring of electrolytes and aggressive treatment is crucial in patients who experience TLS during chemotherapy initiation to prevent complications such as kidney injury, cardiac arrhythmias, and even death. Future studies should focus on prophylactic capacity in patients who have advanced cancer with a high tumor burden.

## References

[1] Lupușoru G, Ailincăi I, Frățilă G (2022). Tumor lysis syndrome: an endless challenge in onco-nephrology. Biomedicines.

[2] Durani U, Hogan WJ (2020). Emergencies in haematology: tumour lysis syndrome. Br J Haematol.

[3] (2024). Tumor lysis syndrome: prevention and treatment. https://www-uptodate-com.acom.idm.oclc.org/contents/tumor-lysis-syndrome-prevention-and-treatment?search=tumor%20lysis%20syndrome&source=search_result&selectedTitle=1%7E107&usage_type=default&display_rank=1.

[4] Rampello E, Fricia T, Malaguarnera M (2006). The management of tumor lysis syndrome. Nat Clin Pract Oncol.

[5] Martelli M, Ferreri A, Agostinelli C, Di Rocco A, Pfreundschuh M, Pileri SA (2013). Diffuse large B-cell lymphoma. Crit Rev Oncol Hematol.

[6] Li S, Young KH, Medeiros LJ (2018). Diffuse large B-cell lymphoma. Pathology.

